# A 17‐year‐old woman with a solitary, mixed squamous cell and glandular papilloma of the bronchus

**DOI:** 10.1002/rcr2.393

**Published:** 2018-11-30

**Authors:** Takeshi Saraya, Masachika Fujiwara, Hirokazu Kimura, Hidefumi Takei, Hajime Takizawa

**Affiliations:** ^1^ Department of Respiratory Medicine Kyorin University School of Medicine Tokyo Japan

**Keywords:** Human papillomavirus, mixed type, solitary endobronchial papilloma, young woman

## Abstract

A 17‐year‐old woman was referred to our hospital due to cough on exertion and right chest pain over the previous two months, together with bloody sputum over the previous week. Chest X‐ray demonstrated a nodule measuring 3 cm in diameter in the right middle lung field. On repeated bronchoscopy, the tumour was recognized as a rapidly growing intra‐bronchial protruded tumour at the orifice of the right B8. Based on a tentative diagnosis of lung cancer, right lower lobectomy was performed. She was diagnosed with mixed squamous cell and glandular papilloma of the bronchus without smoking history and human papillomavirus infection. Solitary endobronchial papillomas are rare but should be considered a differential diagnosis for solitary lung nodule with the potential to develop into carcinoma.

## Introduction

Solitary endobronchial papillomas (SEPs) are extremely rare, accounting for only 0.38% of all lung tumours and approximately 7% of all benign epithelial and mesenchymal lung tumours 1. Here, we present an unexpected case of mixed papilloma in the youngest known patient to date who suffered from chest pain and bloody sputum.

## Case Report

A 17‐year‐old woman visited her local hospital because of cough on exertion and right chest pain over the previous two months, together with bloody sputum over the previous week. At the patient’s local hospital, chest X‐ray depicted an abnormal shadow; therefore, she was referred to our hospital. She was in good health and had no remarkable family history. She had not been exposed to dust or illicit drugs and was not a smoker.

Upon initial assessment, the patient seemed to be well, and her vital signs and physical examinations were normal. Serum laboratory data were normal, including tumour markers such as carcinoembryonic antigen (3.7 ng/mL), carbohydrate antigen 19–9 (16.1 U/mL), sialyl Lewisx‐i antigen (28 U/mL), pro‐gastrin‐releasing peptide (47.8 pg/mL), and soluble interleukin‐2 receptor (307 U/mL). In addition, tests for autoimmune antibodies such as myeloperoxidase‐antineutrophil cytoplasmic antibodies (MPO‐ANCA) and proteinase 3 ANCA were negative.

**Figure 1:**
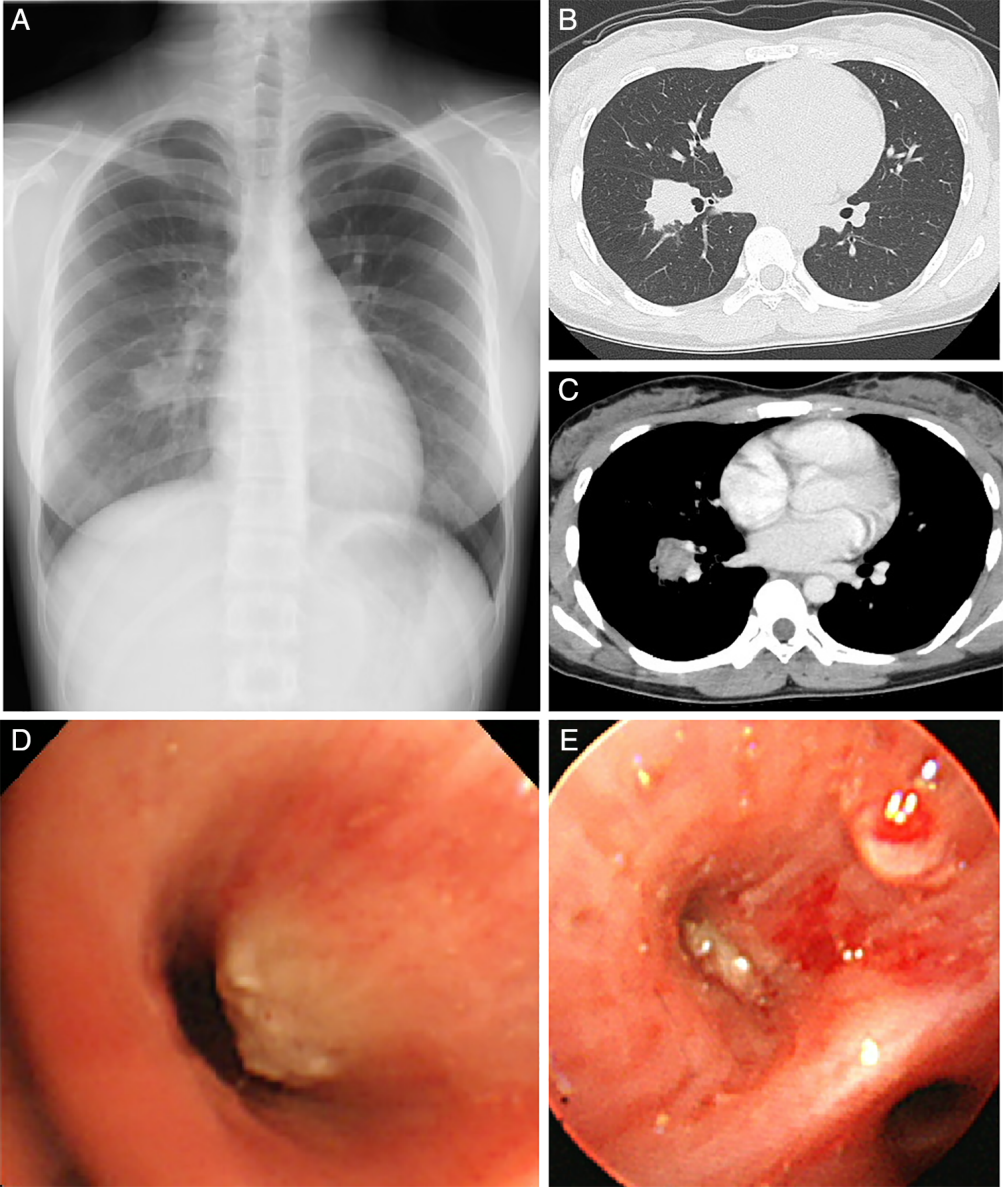
Chest X‐ray on the patient’s first visit to our hospital demonstrated a nodule as large as 3 cm in diameter in the right middle lung fields (A), which was confirmed by contrast‐enhanced thoracic computed tomography as an inhomogeneously enhanced solitary nodule measuring 3 cm in size at the proximal portion of B8/B9 (B, C). Repeated bronchoscopy was performed at 10 hospital days (D) and four weeks (E) after the first visit to our hospital. The tumour compressed the tracheal lumen (D) and then entirely occluded the B8.

**Figure 2:**
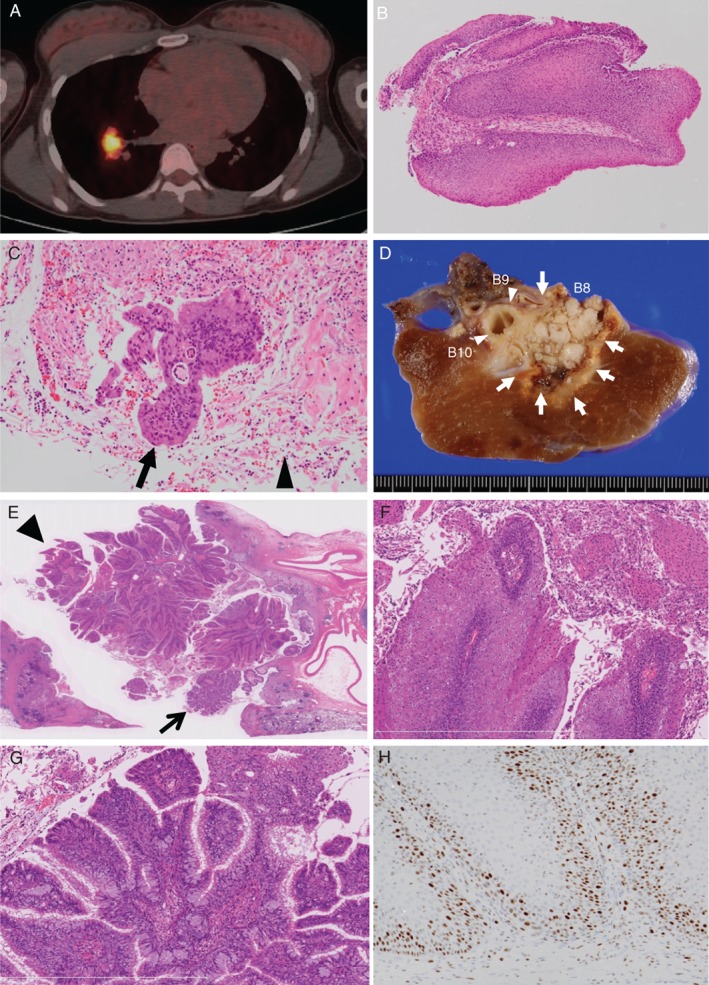
Fluorodeoxyglucose‐positron emission tomography/computed tomography (A) demonstrated that the nodule had an intense standardized uptake value of 11.8. (B, C) Histological findings of the endobronchial biopsied specimens with forceps from the tumour on haematoxylin and eosin (H‐E) staining. The first bronchoscopy demonstrated that a thickened stratified squamous epithelium covered the mucosal surface (B, 40×). A second bronchoscopy, performed three weeks later, showed fragments of ciliated columnar (glandular) epithelium C, 100×, arrow admixed in the abundant squamous epithelium and parakeratotic debris (C, 100×, arrow head). Gross findings of the resected tumour (D, arrows). A whitish tumour occluded the dilated central bronchus (B8) and compressed the adjacent bronchus. Histological findings of the resected tumour (E, F, G, H). The tumour was composed of thick squamous (E, 5×, arrow head) and glandular (E, 5×, arrow) epithelia, both of which proliferated in a papillary pattern. Neither component demonstrated malignant features (F, G 100×). Immunohistochemical staining found an increase of the Ki‐67 labelling index (H, 100×).

On the day of referral to our hospital, chest X‐ray demonstrated a nodule measuring 3 cm in diameter in the right middle lung field (Fig. 1A). At this time, contrast‐enhanced thoracic computed tomography (CT) depicted an inhomogeneously enhanced nodule as large as 3 cm in diameter at the right S8/S9 (Fig. 1B, C). No mediastinal, hilar lymphadenopathies, or other lesions in the lung parenchyma were noted. Fluorodeoxyglucose (FDG) positron emission tomography/computed tomography (PET/CT) depicted the nodule as having intense standardized uptake values of 11.8 (Fig. 2A), suggesting high probability of malignancy.

A bronchoscopy was performed 10 days after the patient’s initial visit. The bronchoscopy showed a protruded white intra‐bronchial tumour at the orifice of the right B8 (Fig. 1D). Haematoxylin and eosin (H&E) staining of the biopsied specimens obtained from the tumour (Fig. 2B, 40×) via endobronchial biopsy with forceps showed that a thickened stratified squamous epithelium covered the mucosal surface. However, these findings did not lead to a diagnosis. Three weeks later, she underwent a second bronchoscopy, which showed that the protruded tumour had progressed and completely occluded the lumen of the B8 (Fig. 1E). H&E staining of endobronchial biopsied specimens with forceps obtained from the tumour showed fragments of ciliated columnar (glandular) epithelium (Fig. 2C, 100×, arrow) admixed in the abundant squamous epithelium and parakeratotic debris (Fig. 2C, 100×, arrow head). Papanicolaou staining of the bronchial washing fluid from the right B8 demonstrated the aggregation of stratified squamous cells with orange‐coloured cytoplasm (data not shown). Repeated bronchoscopy showed no evidence of malignancy or bloody phlegm irrespective of progression of the intra‐bronchial tumour on thoracic CT. However, the intra‐tracheal tumour was considered the source of bloody sputum. Therefore, 40 days after the patient’s first referral to our hospital, she underwent right lower lobe lobectomy via Video‐assisted thoracoscopic surgery.

A resection of the right lower lobe (14 × 11 × 5 cm) indicated a white‐coloured papillary tumour measuring 4 cm in diameter located at the proximal portion of the dilated bronchus (Fig. 2D), which consequently leads to the right B8a. H&E staining showed that the tumour was composed of thick squamous (Fig. 2E, 5×, arrow head) and glandular (Fig. 2E, 5×, arrow) epithelia, both of which proliferated in a papillary pattern along with inflamed fibrovascular cores. The former (squamous) was predominant, and neither component demonstrated malignant features (Fig. 2F, G 100×). Immunohistochemical staining for Ki‐67 found strong positivity up to the basal half of the squamous component (Fig. 2H, 100×). This increase of the positive index indicated active proliferation of the tumour. Upon further histological analysis, our case showed a negative result both in polymerase chain reaction for human papillomavirus (HPV) and immunohistochemical staining with p16^Ink4a^ **,** a surrogate molecular marker of HPV. Finally, she was diagnosed with mixed squamous cell and glandular papilloma of the bronchus without HPV infection.

## Discussion

SEPs are extremely rare, accounting for only 0.38% of all lung tumours and approximately 7% of all benign epithelial and mesenchymal lung tumours 1. SEPs are classified into three types: (1) squamous cell papilloma; (2) glandular papilloma; and (3) mixed squamous cell and glandular papilloma (mixed papilloma). Among those categories, the latter is the rarest of the SEPs and accounts for only 16% of all cases 2. To the best of our knowledge, only 22 cases of mixed papilloma have been reported in the English‐language literature 3, and the present case involves the youngest patient reported to date.

Previous reports have described that solitary squamous papillomas are found predominantly in middle‐aged men, generally in those with a past or current history of tobacco smoking. Glandular and mixed papillomas occur in older men and women and are less related to tobacco smoking than other types of papilloma. However, other studies have reported different trends, such as the proportion of smokers and median age being highest in mixed papillomas and glandular papilloma, respectively. From this perspective, the present case is quite atypical for a case of mixed papilloma in that a young female patient without a history of smoking developed a mixed papilloma. Squamous cell and mixed papilloma have the potential to develop into carcinoma. There are only five cases of mixed papillomas with malignant features in previous reports 4. Therefore, progression of a tumour over a time period as short as in the present case might require surgical resection for a definite diagnosis.

Thoracic CT and PET/CT findings of SEPs have been reported only rarely; however, a few cases have shown increased FDG uptake in the tumour. This might correspond to active cell proliferation of the papilloma, as was found in the present case. It is well known that FDG‐PET has a sensitivity of 97% and a specificity of 78% in the characterization of malignant solitary pulmonary nodules that are >10 mm in diameter. A standardized uptake value (SUV) > 2.5 is generally considered to be highly suggestive of malignancy or active inflammation. In this regard, the radiological diagnosis of papilloma might be difficult to differentiate from that of malignancy.

The putative risk factors for malignant transformation are smoking and HPV infection serotypes 16, 18, and 31/33/35. These findings were compatible with previous reports that HPV deoxyribonucleic acid (DNA) is detected in most cases of squamous cell papilloma, while HPV DNA has not been found in mixed papilloma of the lung 4. Interestingly, Miyoshi et al. recently reported the first case of mixed papilloma positive for p16^Ink4a^ 5. This finding requires further index cases in order to understand the pathogenic role of HPV both in the generation of mixed papilloma and in its vulnerability for malignancy.

The present study reported a case of pulmonary papilloma occurring in the youngest known patient to date. In this case, the papilloma presented as bloody phlegm with a solitary pulmonary nodule. The differentiation of papilloma from malignancy is a pivotal issue for physicians and requires clinical and/or radiological examination together with sufficient surgical intervention.

### Disclosure Statement

Appropriate written informed consent was obtained for publication of this case report and accompanying images.
